# Supplementary material for: Next Generation CD44v6-Specific CAR-NK Cells Effective against Triple Negative Breast Cancer
Source: Int J Mol Sci. 2023 May 20;24(10):9038. doi: 10.3390/ijms24109038 (PMC10218876; doi:10.3390/ijms24109038)
Supplement: Supplementary file 1 [file ijms-24-09038-s001.zip › ijms-2341370-supplementary.pdf]

Supplementary material

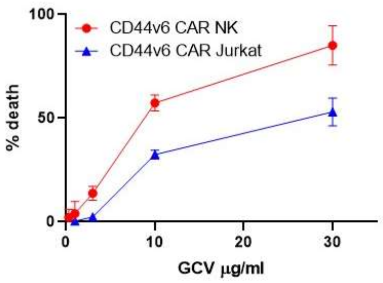

Supplementary Figure S1 | Suicide gene allows removal of CD44v6 CAR expressing cells. In order to validate the suicide gene we tested transduced Jurkat cells and primary NK cells with increasing does of Gancyclovir (GCV) for 48 hrs before determination of cell death by propidium iodide staining. All transduced cell lines showed significant sensitivity to GCV at therapeutically relevant doses. Results are shown as specific death of CAR-expressing cells relative to a positive control. Assay done in triplicate.

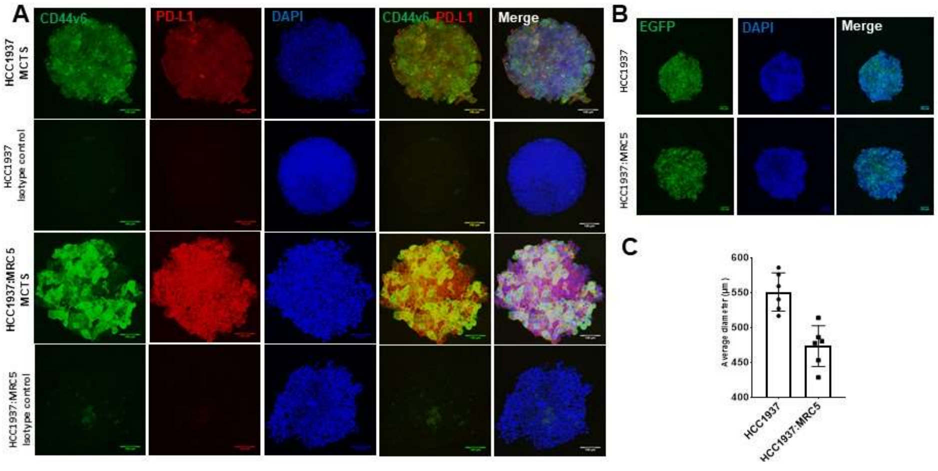

Supplementary Figure S2 | Confocal microscopy and characteristics of 3D TNBC tumor models. TNBC tumor spheroids with roughly 500 μm in diameter were stained for their respective markers and imaged in a confocal microscope at x10 or x20 magnification, excited with a solid-state laser (405, 488 and 635 nm). Isotype controls of the antibodies are shown directly beneath the measured sample spheroids. **A)** The CAR target molecule CD44v6 (green) and PD-L1 (red) expression can be seen evenly across the surface of the measured tumor spheroids in both TNBC spheroid models. **B)** HCC1937 and HCC1937:MRC5 tumor spheroids at x10 magnification showing the EGFP expression of transduced HCC1937 cells in the tumor spheroid model. All EGFP expressing cells are also

expressing nanoluciferase. In the HCC1937:MRC5 co-culture model an even dispersion of EGFP expressing HCC1937 cells can be seen across the surface of the tumor spheroid. The MRC5 fibroblasts seem to arrange themselves evenly across the surface fulfilling their tumor cell supportive role. **C)** Graphical depiction of the average diameter in μm of HCC1937 spheroids and HCC1937:MRC5 (1:1) co-culture spheroids with a fixed total cell number of $2x10^3$, the average diameter was calculated from the measured area of the MCTS.
